# Premenopausal endogenous steroid hormones and breast cancer risk: results from the Nurses' Health Study II

**DOI:** 10.1186/bcr3394

**Published:** 2013-03-06

**Authors:** Renée T Fortner, A Heather Eliassen, Donna Spiegelman, Walter C Willett, Robert L Barbieri, Susan E Hankinson

**Affiliations:** 1 Channing Division of Network Medicine, Department of Medicine, Brigham and Women's Hospital, 181 Longwood Ave., Boston, MA 02115, USA; 2 Harvard School of Public Health, 677 Huntington Ave., Boston, MA 02115, USA; 3 Harvard Medical School, 25 Shattuck St., Boston, MA 02115, USA; 4 Department of Obstetrics, Gynecology, and Reproductive Medicine, Brigham and Women's Hospital, 75 Francis St., Boston, MA 02115, USA; 5 Division of Biostatistics and Epidemiology, University of Massachusetts, 715 N. Pleasant St., Amherst, MA 01002, USA

## Abstract

**Introduction:**

Prior research supports an association between endogenous sex steroids and breast cancer among postmenopausal women; the association is less clear among premenopausal women.

**Methods:**

We evaluated the associations between estrogens, androgens, progesterone and sex hormone binding globulin (SHBG) and breast cancer in a nested case-control study in the Nurses' Health Study II. Between 1996 and 1999, 29,611 participants provided blood samples; 18,521 provided samples timed in early follicular and mid-luteal phases of the menstrual cycle. A total of 634 women, premenopausal at blood collection, developed breast cancer between 1999 and 2009 and were matched to 1,264 controls (514 cases and 1,030 controls with timed samples). We used conditional logistic regression controlling for breast cancer risk factors for overall analyses; unconditional logistic regression additionally controlling for matching factors was used for subgroup analyses.

**Results:**

In analyses of premenopausal estrogens including breast cancers diagnosed both before and after menopause, there was no association between follicular estradiol, estrone and free estradiol and risk of either total or invasive breast cancer. Luteal estradiol was positively associated with estrogen receptor positive (ER+)/progesterone receptor positive (PR+) cancers (5^th ^vs. 1^st ^quintile odds ratio (OR): 1.7 (95% confidence interval (CI): 1.0 to 2.9), *P*_trend _= 0.02). Luteal estrone, free estradiol and progesterone were not associated with risk. Androgens were suggestively or significantly associated with risk when the sample was restricted to invasive tumors (for example, testosterone: OR: 1.4 (1.0 to 2.0), *P*_trend _= 0.23) and ER+/PR+ disease (testosterone: OR: 1.7 (1.1 to 2.6) *P*_trend _= 0.10; dehydroepiandrosterone sulfate (DHEAS) OR: 1.3 (0.8 to 2.0) *P*_trend _= 0.05). SHBG was not associated with breast cancer risk. The results varied by menopausal status at diagnosis, with follicular estradiol suggestively positively associated with breast cancers in women premenopausal at diagnosis (OR: 1.1 (0.9 to 1.3) and significantly inversely associated with postmenopausal disease (OR: 0.6 (0.4 to 0.9); *P*_heterogeneity _< 0.01).

**Conclusions:**

Androgens were associated with modestly increased risk of breast cancer in this population, with stronger associations for invasive and ER+/PR+ disease. Luteal phase estradiol levels were suggestively associated with ER+/PR+ tumors but no other strong associations were observed with estrogens. Associations with follicular phase estrogens may vary by menopausal status at diagnosis, but case numbers were limited. Additional studies to confirm the role of premenopausal hormones in the etiology of both premenopausal and postmenopausal breast cancer are needed.

## Introduction

Experimental data support an association between estrogens and breast cancer risk, and suggest that androgens also may alter risk. Estrogens are hypothesized to act through cell proliferation, decreased apoptosis and possibly DNA damage [1]. The role of androgens is more complex, with both inhibitory [2,3] and proliferative [3] effects previously shown in laboratory studies.

In epidemiologic studies, circulating sex steroid hormones are well confirmed to increase risk of postmenopausal breast cancer [4], but data on these associations in premenopausal women are sparse. A major challenge in evaluating estrogens and disease risk in premenopausal women is the fluctuation in estrogen levels across the menstrual cycle. To date there have been seven small (case n < 100) [5-11] and three larger prospective studies (case n range = 197 to 285) [12-15], including our own [13,14], of the association between circulating premenopausal hormones and breast cancer risk. A positive association between circulating estrogens and breast cancer risk was suggested in four of the smaller studies [5,6,9,11], but only one of the larger studies [13] (a subset of the population included in this analysis). Data are more consistent for circulating androgens, with higher premenopausal androgen levels associated with increased risk [5,8,12-15]. Only three prior studies, including our initial reports, evaluated results by tumor hormone receptor status [13-15], a particularly important consideration given the substantial data suggesting heterogeneity in risk factor associations by tumor hormone receptor status [16-19].

Given the limitations of the prior literature, we conducted a nested case-control study in the Nurses' Health Study II (NHSII) to examine associations among breast cancer, overall and by hormone receptor subtype, and plasma estrogens and progesterone by menstrual cycle phase, as well as androgens and sex hormone binding globulin (SHBG). The current analyses include six additional years of follow-up and more than double the number of cases compared to our initial evaluations [13,14].

## Materials and methods

The NHSII began in 1989 when 116,430 female registered nurses, ages 25 to 42 y, completed a mailed questionnaire. The participants have been followed biennially to update exposure information and ascertain disease diagnoses. Between 1996 and 1999 the blood cohort (*n *= 29,611), a subset of the study population, ages 32 to 54 y, provided blood and urine samples.

Women who had not used oral contraceptives, been pregnant or breastfed within the past six months (*n *= 18,521) provided blood samples timed within the early follicular (days 3 to 5 of the menstrual cycle) and mid-luteal (seven to nine days prior to the expected start of the next menstrual cycle) phases of the menstrual cycle; the remainder provided single untimed samples. Participants were mailed a sample collection kit with detailed instructions and a questionnaire. Women providing timed samples separated, then froze the follicular plasma 8 to 24 hours after collection, and stored this sample in their home freezer to be shipped with the luteal phase sample. After collection of the luteal sample, or untimed sample, samples were sent with an ice pack via overnight courier to our laboratory where they were processed and have been subsequently stored in continuously monitored liquid nitrogen freezers at ≤ -130°C. Approximately 93% of samples were received within 26 hours of collection. The stability of sex steroids using these blood collection methods has been previously validated [20,21]. A total of 97% of the women providing timed samples returned a postcard with the start date of the next menstrual cycle, which allows for precise calculation of luteal phase collection timing using backward dating. Follow-up of the blood cohort through the 2009 cycle is 94.5%. This investigation was approved by the Institutional Review Board of the Brigham and Women's Hospital. Informed consent was implied by receipt of completed questionnaires and blood samples.

### Case and control selection

Participants report disease status on the biennial NHSII questionnaires. Cases included in this analysis were premenopausal at blood collection, reported no prior cancer diagnosis, and were diagnosed with breast cancer through the 2009 biennial questionnaire; 634 breast cancer cases were identified. Breast cancer cases were confirmed through medical record review by a study physician; invasive vs. *in situ *and hormone receptor status was abstracted from the medical record. Of the 634 cases identified, 607 (95.7%) were confirmed through medical record review; the remaining 27 cases (4.3%) were verbally confirmed by the nurse but medical records were not available. Given the high confirmation rate by medical record for breast cancer in this cohort (99%), all cases are included in this analysis. Among the 634 cases, 428 were invasive and 295 were ER+/PR+. A total of 425 women were premenopausal at diagnosis, while 144 were postmenopausal at diagnosis (timed and untimed samples).

Cases were diagnosed an average of 6.2 years after blood draw (range 1 month to 13 years). Each case was matched to two controls on age at blood draw (± 2 years), menopausal status at the time of the case's diagnosis, race/ethnicity (African-American, Asian, Hispanic, Caucasian, other), luteal day of menstrual cycle (defined as the date of a woman's next period minus the date of the luteal blood draw, ± 1 day; timed samples only), and for each blood collection, fasting status (< 2, 2 to 4, 5 to 7, 8 to 11, and 12+ hours), month and year (± 2 months) and time of day of blood collection (± 2 hours).

### Laboratory assays

Case-control sets, as well as follicular and luteal samples from the same person, were assayed together. Samples were ordered randomly within a set, and laboratories were masked to both case-control status and repeated samples within woman. Samples were assayed for estrogens and testosterone in five batches at either Quest Diagnostics (San Juan Capistrano, CA, USA) by radioimmunoassay preceded by organic extraction and celite chromatography (batches 1 and 2) [22-24] or the Mayo Clinic (Rochester, MN, USA) by liquid chromatography-tandem mass spectrometry (batches 3 to 5). Results from a pilot study showed a correlation of > 0.9 between these two methods. The first four of five total batches of progesterone, SHBG, and dehydroepiandrosterone sulfate (DHEAS) were assayed at the Royal Marsden Hospital (London, UK) by chemiluminescence immunoassay and an Immulite autoanalyzer (Diagnostic Products, Gwynedd, UK). Batch 5 of progesterone and SHBG were assayed at Massachusetts General Hospital (Boston, MA, USA); progesterone was assayed using automated immunoassay and SHBG using an automated two-site chemiluminescent immunometric assay (ARCHITECT^R^, Abbott Diagnostics, Chicago, IL). Batch 5 of DHEAS was assayed at the Mayo Clinic by a solid-phase, chemiluminescent enzyme immunoassay (Siemens Healthcare Diagnostics, Deerfield, IL, USA). Masked replicate quality control samples (10% of the samples) were included in each batch to assess coefficients of variation (CVs). Average CVs within batch ranged from 5% for DHEAS to 14% for SHBG, with the exception of progesterone for which the CV was 17% in one batch (CVs for progesterone 7 to 15% in remaining batches). Free estradiol and free testosterone were calculated using the formula described by Södergard *et al. *[25].

### Covariate data

Lifestyle factors and other exposures were collected on the biennial questionnaire, as well as a questionnaire completed at the time of blood collection. Covariates included in this analysis were (year of data collection): age at menarche (1989), height and weight at age 18 (1989; used to calculate body mass index (BMI), kg/m^2^), parity (biennially), age at first birth (biennially), family history of breast cancer (1989 and 1997; mother and/or sister), history of benign breast disease (biennially).

### Statistical methods

Statistical outliers were identified on the log_2 _scale for each hormone by menstrual phase using the extreme Studentized deviate many-outlier procedure [26], with as many as 11 values excluded (luteal free estradiol). We created quintiles of hormones and SHBG using the control distributions for overall analyses and quartiles based on the control distribution for analyses of premenopausal vs. postmenopausal breast cancer. To adjust for between-batch differences in hormone distributions, we used an average batch recalibration approach [27], taking into account batch, age and BMI, as well as luteal day of collection and ovulatory status in the cycle of collection for luteal samples, and follicular day of collection for follicular samples. Results using recalibrated data or using batch-specific quantile cutpoints were comparable, hence only recalibrated data are presented here. For estradiol, free estradiol and estrone, menstrual cycle phase-specific quintile cut-points were used (for example, follicular, luteal phase). Estimates for doubling of hormone concentrations were assessed modeling the data continuously after log_2 _transformation.

We used conditional logistic regression to estimate relative risks (RR) and 95% confidence intervals (95% CI) in analyses including all cancers. For subgroup and stratified analyses, we used unconditional logistic regression controlling for matching factors. Multivariable models were adjusted for BMI at age 18, age at menarche, age at first birth and parity, family history of breast cancer, and history of benign breast disease. Tests for trend were conducted using quantile medians and *P*-values were calculated with the Wald statistic. Heterogeneity by hormone receptor subtype was evaluated using polytomous logistic regression [28], using the likelihood ratio test to compare a model with separate slopes by case status (that is, ER+/PR+, ER-/PR-, control) to a model with the same slope for each case group. We conducted analyses correcting for measurement error, using samples from a within-person reproducibility study [20], to correct for and explore the effects of random within-person measurement error [29,30]. We assessed whether the associations between hormone levels and risk varied by menopausal status at diagnosis, age at blood draw, BMI, duration of oral contraceptive use, or menstrual cycle length and pattern by assessing the statistical significance of an interaction term included in our models. We cross-classified estradiol levels by phase of the menstrual cycle; women with low levels (below the median) in both the follicular and luteal phase were used as the referent group. Finally, we conducted secondary analyses restricted to women with ovulatory cycles (defined by mid-luteal progesterone > 400 ng/dL) at the time of blood collection, as well as to cases diagnosed (and controls selected) at least two years after blood collection.

All statistical tests are two-sided; *P *< 0.05 was used to define statistical significance. Analyses were conducted in SAS v. 9.2 (Cary, NC, USA).

## Results

Cases and controls were similar with regards to most breast cancer risk factors (Table 1), including age at menarche, parity, age at first birth and BMI. However, cases were more likely than controls to have a history of benign breast disease and family history of breast cancer. Participants were predominantly premenopausal at diagnosis (67%).

**Table 1:** Age-adjusted characteristics of study participants by case/control status: Nurses' Health Study, 1999 to 2009

	Cases (*n *= 634)	Controls (*n *= 1,264)
Age at blood collection*	43.5 (4.0)	43.3 (3.9)
BMI (kg/m^2^) at blood collection	24.9 (4.8)	25.7 (5.9)
BMI (kg/m^2^) at age 18	20.8 (2.9)	21.1 (3.0)
Age at menarche	12.4 (1.3)	12.5 (1.4)
Parous, %	80	81
Age at first birth**	27.4 (4.5)	26.7 (4.5)
Luteal day, prior to next cycle	8.4 (3.1)	8.4 (2.9)
Follicular day, after cycle start	4.0 (0.8)	4.0 (1.3)
		
Menopausal status at diagnosis		
Premenopausal, %	67	69
Postmenopausal, %	23	23
Perimenopausal/Unknown, %	10	8
Family history of breast cancer, %	18	10
History of benign breast disease, %	22	14

Values are means (SD) or percentages and are standardized to the age distribution of the study population. *Value is not age adjusted. **Among parous women.

There were no associations between follicular estradiol, estrone and free estradiol and breast cancer risk overall, or by invasive, *in situ *or ER+/PR+ subgroup (Table 2). Luteal estradiol was not associated with all cancers combined, invasive or *in situ *cancers, but was significantly, positively associated with ER+/PR+ cancers (5^th ^vs. 1^st ^quintile OR: 1.7, 95% CI: 1.0 to 2.9, *P*_trend _= 0.02). After exclusion of women anovulatory in the cycle of blood collection, the OR was somewhat attenuated and no longer statistically significant (5^th ^vs. 1^st ^quintile OR: 1.5, 95% CI: 0.8 to 2.7, data not shown). No significant associations were observed for luteal estrone, free estradiol or progesterone overall or by subgroup.

**Table 2:** Premenopausal estrogens and progesterone and breast cancer risk: Nurses' Health Study II, 1999 to 2009

	1st Quintile	2nd Quintile	3rd Quintile	4th Quintile	5th Quintile	
	OR (95% CI)	OR (95% CI)	OR (95% CI)	OR (95% CI)	OR (95% CI)	*P*_trend_
**FOLLICULAR**						
**Estradiol**						
Cutpoints, pg/mL	< 29	≥ 29 to 40.9	≥ 41 to 51.9	≥ 52 to 73.9	≥ 74	
No. cases/No. controls*	84/179	93/183	84/179	109/186	92/182	
All cancers	1.0 (referent)	1.1 (0.7 to 1.6)	1.0 (0.7 to 1.5)	1.2 (0.8 to 1.7)	1.0 (0.7 to 1.5)	0.76
Invasive	1.0 (referent)	1.2 (0.8 to 1.8)	1.0 (0.6 to 1.6)	1.4 (0.9 to 2.1)	1.0 (0.7 to 1.6)	0.70
ER+/PR+	1.0 (referent)	1.1 (0.7 to 1.8)	0.9 (0.5 to 1.5)	1.5 (0.9 to 2.4)	1.0 (0.6 to 1.6)	0.68
*In situ*	1.0 (referent)	1.0 (0.5 to 1.8)	1.1 (0.6 to 1.9)	0.8 (0.5 to 1.5)	1.1 (0.6 to 2.0)	0.81
**Free estradiol**						
Cutpoints, pg/mL	< 0.39	≥ 0.39 to 0.519	≥ 0.52 to 0.669	≥ 0.67 to 0.909	≥ 0.91	
No. cases/No. controls	90/178	80/175	104/179	96/176	77/178	
All cancers	1.0 (referent)	0.9 (0.6 to 1.4)	1.1 (0.7 to 1.5)	1.1 (0.8 to 1.7)	0.8 (0.5 to 1.2)	0.48
Invasive	1.0 (referent)	1.0 (0.6 to 1.5)	1.0 (0.6 to 1.5)	1.2 (0.8 to 1.9)	0.8 (0.5 to 1.2)	0.50
ER+/PR+	1.0 (referent)	1.1 (0.7 to 1.9)	1.2 (0.7 to 2.0)	1.3 (0.8 to 2.1)	0.7 (0.4 to 1.2)	0.35
*In situ*	1.0 (referent)	0.9 (0.5 to 1.7)	1.2 (0.7 to 2.1)	0.8 (0.5 to 1.6)	0.9 (0.5 to 1.7)	0.77
**Estrone**						
Cutpoints, pg/mL	< 30	≥ 30 to 36.9	≥ 37 to 43.9	≥ 44 to 55.9	≥ 56	
No. cases/No. controls	93/184	86/192	91/176	111/184	88/184	
All cancers	1.0 (referent)	0.9 (0.6 to 1.2)	1.0 (0.7 to 1.4)	1.2 (0.8 to 1.7)	1.0 (0.7 to 1.4)	0.62
Invasive	1.0 (referent)	0.8 (0.5 to 1.3)	1.1 (0.7 to 1.7)	1.1 (0.7 to 1.7)	0.9 (0.6 to 1.3)	0.92
ER+/PR+	1.0 (referent)	1.2 (0.7 to 2.0)	1.3 (0.8 to 2.2)	1.6 (1.0 to 2.7)	1.2 (0.7 to 2.0)	0.32
*In situ*	1.0 (referent)	1.1 (0.6 to 2.0)	0.8 (0.4 to 1.5)	1.5 (0.8 to 2.6)	1.3 (0.7 to 2.3)	0.22
**LUTEAL**						
**Estradiol**						
Cutpoints, pg/mL	< 91	≥ 91 to 116.9	≥ 117/146.9	≥ 147 to 186.9	≥ 187	
No. cases/No. controls	90/197	72/187	123/187	113/195	81/193	
All cancers	1.0 (referent)	0.8 (0.6 to 1.3)	1.5 (1.0 to 2.2)	1.3 (0.9 to 1.9)	0.9 (0.6 to 1.4)	0.65
Invasive	1.0 (referent)	1.0 (0.6 to 1.6)	1.9 (1.3 to 2.9)	1.5 (1.0 to 2.3)	1.3 (0.8 to 2.0)	0.08
ER+/PR+	1.0 (referent)	1.2 (0.7 to 2.2)	2.5 (1.5 to 4.2)	1.9 (1.1 to 3.2)	1.7 (1.0 to 2.9)	0.02
*In situ*	1.0 (referent)	0.6 (0.3 to 1.2)	1.1 (0.6 to 1.9)	1.0 (0.6 to 1.7)	0.5 (0.3 to 0.9)	0.17
**Free estradiol**						
Cutpoints, pg/mL	< 1.20	≥ 1.20 to 1.49	≥ 1.50 to 1.89	≥ 1.90 to 2.39	≥ 2.40	
No. cases/No. controls	94/188	95/190	93/188	100/189	87/189	
All cancers	1.0 (referent)	1.1 (0.7 to 1.6)	1.0 (0.7 to 1.5)	1.1 (0.8 to 1.6)	1.0 (0.7 to 1.5)	0.99
Invasive	1.0 (referent)	1.2 (0.8 to 1.8)	1.3 (0.9 to 2.0)	1.2 (0.8 to 1.9)	1.2 (0.8 to 1.9)	0.36
ER+/PR+	1.0 (referent)	1.3 (0.8 to 2.2)	1.5 (0.9 to 2.6)	1.5 (0.9 to 2.6)	1.3 (0.7 to 2.1)	0.28
*In situ*	1.0 (referent)	0.9 (0.5 to 1.5)	0.6 (0.3 to 1.1)	0.8 (0.5 to 1.5)	0.7 (0.4 to 1.4)	0.32
**Estrone**						
Cutpoints, pg/mL	< 61	≥ 61 to 74.9	≥ 75 to 91.9	≥ 92 to 113.9	≥ 114	
No. cases/No. controls	121/198	84/201	90/206	92/201	113/199	
All cancers	1.0 (referent)	0.7 (0.5 to 1.0)	0.8 (0.5 to 1.1)	0.8 (0.5 to 1.1)	0.9 (0.7 to 1.3)	0.89
Invasive	1.0 (referent)	0.7 (0.5 to 1.1)	0.8 (0.5 to 1.2)	0.8 (0.5 to 1.1)	1.1 (0.7 to 1.6)	0.67
ER+/PR+	1.0 (referent)	0.5 (0.3 to 0.8)	0.8 (0.5 to 1.2)	0.8 (0.5 to 1.3)	0.9 (0.6 to 1.5)	0.87
*In situ*	1.0 (referent)	0.7 (0.4 to 1.2)	0.8 (0.4 to 1.3)	0.8 (0.5 to 1.4)	0.8 (0.4 to 1.3)	0.55
**Progesterone**						
Cutpoints, ng/dL	< 743	≥ 743 to 1202.9	≥ 1203 to 1600.9	≥ 1601 to 2170.9	≥ 2171	
No. cases/No. controls	113/204	82/199	86/204	113/200	107/202	
All cancers	1.0 (referent)	0.7 (0.4 to 1.0)	0.8 (0.5 to 1.1)	1.0 (0.7 to 1.4)	0.9 (0.6 to 1.4)	0.82
Invasive	1.0 (referent)	0.7 (0.4 to 1.0)	0.7 (0.5 to 1.1)	1.0 (0.7 to 1.5)	1.0 (0.6 to 1.5)	0.91
ER+/PR+	1.0 (referent)	0.6 (0.4 to 1.0)	0.7 (0.4 to 1.1)	1.0 (0.6 to 1.6)	0.9 (0.6 to 1.5)	0.74
*In situ*	1.0 (referent)	0.7 (0.4 to 1.2)	0.8 (0.4 to 1.4)	0.8 (0.4 to 1.4)	0.8 (0.5 to 1.5)	0.43

All cancers: Conditional logistic regression for all cancers controlling for age at menarche, parity/age at first birth, BMI at age 18, family history of breast cancer, history of benign breast disease. Invasive, ER+/PR+, *in situ *tumors: Unconditional logistic regression for invasive, ER+/PR+, and *in situ *disease controlling for factors listed above and matching factors. *No. cases/No. controls are for all cancers; Overall No. cases/No. controls for invasive and ER+/PR+ cancers are in Table 4.

Testosterone was not associated with breast cancer overall but was associated with invasive (5^th ^vs. 1^st ^OR: 1.4, 95% CI: 1.0 to 2.0, *P*_trend _= 0.23) and ER+/PR+ disease (5^th ^vs. 1^st ^quintile OR: 1.7, 95% CI: 1.1 to 2.6, *P*_trend _= 0.10) (Table 3). Free testosterone was similarly associated with ER+/PR+ disease (5^th ^vs. 1^st ^OR: 1.5, 95% CI: 1.0 to 2.2, *P*_trend _= 0.09). Tests for trend suggested a positive association between DHEAS and risk of ER+/PR+ breast cancer (*P*_trend _= 0.05), but the effect estimates did not reach statistical significance (5^th ^vs. 1^st ^quintile OR: ER+/PR+ = 1.3, 95% CI: 0.8 to 2.0). SHBG was not associated with breast cancer risk.

**Table 3:** Premenopausal androgens and SHBG and breast cancer risk: Nurses' Health Study II, 1999 to 2009

	1st Quintile	2nd Quintile	3rd Quintile	4th Quintile	5th Quintile	
	OR (95% CI)	OR (95% CI)	OR (95% CI)	OR (95% CI)	OR (95% CI)	*P*_trend_
**Testosterone**						
Cutpoints, ng/dL	< 19	≥ 19 to 22.9	≥ 23 to 27.9	≥ 28 to 34.9	≥ 35	
No. cases/No. controls*	126/259	122/244	107/246	118/240	150/256	
All cancers	1.0 (referent)	1.0 (0.7 to 1.4)	0.9 (0.6 to 1.2)	0.9 (0.7 to 1.3)	1.2 (0.9 to 1.7)	0.32
Invasive	1.0 (referent)	1.2 (0.8 to 1.7)	1.0 (0.7 to 1.4)	0.9 (0.6 to 1.3)	1.4 (1.0 to 2.0)	0.23
ER+/PR+	1.0 (referent)	1.5 (1.0 to 2.3)	1.1 (0.7 to 1.8)	1.0 (0.6 to 1.5)	1.7 (1.1 to 2.6)	0.10
*In situ*	1.0 (referent)	0.9 (0.5 to 1.5)	0.9 (0.5 to 1.5)	1.2 (0.7 to 1.9)	1.0 (0.6 to 1.7)	0.64
**Free testosterone**						
Cutpoints, ng/dL	< 0.14	≥ 0.14 to 0.179	≥ 0.18 to 0.239	≥ 0.24 to 0.309	≥ 0.31	
No. cases/No. controls	130/248	108/246	137/249	100/246	142/248	
All cancers	1.0 (referent)	0.8 (0.6 to 1.1)	1.0 (0.7 to 1.4)	0.8 (0.6 to 1.1)	1.1 (0.8 to 1.5)	0.61
Invasive	1.0 (referent)	0.9 (0.6 to 1.3)	1.1 (0.8 to 1.6)	0.9 (0.6 to 1.3)	1.1 (0.8 to 1.6)	0.49
ER+/PR+	1.0 (referent)	1.0 (0.6 to 1.5)	1.1 (0.7 to 1.7)	1.0 (0.6 to 1.5)	1.5 (1.0 to 2.2)	0.09
*In situ*	1.0 (referent)	0.9 (0.6 to 1.6)	1.1 (0.7 to 1.8)	0.7 (0.4 to 1.3)	1.3 (0.8 to 2.1)	0.47
**DHEAS**						
Cutpoints, μg/dL	< 55	≥ 55 to 76.9	≥ 77 to 100.9	≥ 101 to 135.9	≥ 136	
No. cases/No. controls	123/249	96/253	136/250	146/251	127/251	
All cancers	1.0 (referent)	0.8 (0.5 to 1.1)	1.1 (0.8 to 1.6)	1.2 (0.9 to 1.6)	1.0 (0.7 to 1.4)	0.30
Invasive	1.0 (referent)	0.8 (0.5 to 1.1)	1.1 (0.8 to 1.6)	1.1 (0.8 to 1.6)	1.1 (0.7 to 1.5)	0.29
ER+/PR+	1.0 (referent)	0.9 (0.5 to 1.4)	1.4 (0.9 to 2.2)	1.5 (1.0 to 2.2)	1.3 (0.8 to 2.0)	0.05
*In situ*	1.0 (referent)	0.8 (0.5 to 1.4)	1.0 (0.6 to 1.7)	1.3 (0.8 to 2.2)	0.9 (0.5 to 1.6)	0.62
**SHBG**						
Cutpoints, nmol/mL	< 40	≥ 40 to 54.9	≥ 55 to 70.9	≥ 71 to 91.9	≥ 92	
No. cases/No. controls	110/253	117/245	131/249	136/249	130/250	
All cancers	1.0 (referent)	1.1 (0.8 to 1.5)	1.2 (0.8 to 1.6)	1.2 (0.9 to 1.7)	1.2 (0.8 to 1.6)	0.23
Invasive	1.0 (referent)	1.2 (0.8 to 1.8)	1.4 (1.0 to 2.0)	1.3 (0.9 to 1.9)	1.3 (0.9 to 1.9)	0.13
ER+/PR+	1.0 (referent)	1.1 (0.7 to 1.7)	1.3 (0.8 to 2.0)	1.3 (0.9 to 2.0)	1.2 (0.8 to 1.8)	0.35
*In situ*	1.0 (referent)	0.8 (0.5 to 1.4)	0.9 (0.5 to 1.5)	1.0 (0.6 to 1.7)	0.8 (0.5 to 1.4)	0.72

All cancers: Conditional logistic regression for all cancers controlling for age at menarche, parity/age at first birth, BMI at age 18, family history of breast cancer, history of benign breast disease. Invasive, ER+/PR+, *in situ *tumors: Unconditional logistic regression for invasive, ER+/PR+, and *in situ *disease controlling for factors listed above and matching factors. *No. cases/No. controls are for all cancers; Overall No. cases/No. controls for invasive and ER+/PR+ cancers are in Table 4.

To examine the importance of menopausal status at diagnosis, we repeated the analyses stratifying by menopausal status at diagnosis (premenopausal: case *n *= 425 overall; *n *= 373 with timed samples; postmenopausal: case *n *= 144 overall; *n *= 104 with timed samples) (Table 4, Additional file 1). For the estrogens, among women premenopausal at diagnosis, a doubling of follicular estradiol was suggestively positively associated with invasive and ER+/PR+ breast cancer (invasive, OR: 1.2, 95% CI: 1.0 to 1.4; ER+/PR+, OR: 1.2 95% 0.9 to 1.5). In contrast, for women postmenopausal at diagnosis, we observed a significant inverse association (all cancers, OR: 0.6, 95% CI: 0.4 to 0.9; invasive, OR: 0.6, 95% CI: 0.4 to 0.9; ER+/PR+, OR: 0.6, 95% CI: 0.4 to 0.9; *P*_heterogeneity _all ≤ 0.01). Associations for follicular free estradiol were similar to those for total estradiol; follicular estrone was not associated with disease in pre- or postmenopausal women. Associations with a doubling of luteal estradiol and free estradiol were somewhat stronger in premenopausal disease as compared to postmenopausal disease (that is, luteal estradiol, invasive premenopausal disease OR: 1.2, 95% CI: 1.0 to 1.6; invasive postmenopausal disease OR: 1.1, 95% CI: 0.7 to 1.6). However, findings for luteal estradiol were attenuated after restricting them to women with ovulatory cycles at blood collection (Additional file 1, Table S1). For progesterone, there was no association for premenopausal cancer (invasive, OR: 1.1, 95% CI: 0.9 to 1.2) and an inverse association for postmenopausal cancer (invasive, OR: 0.8, 95% CI: 0.7 to 1.0), though these differences were not statistically significant (*P*_het _= 0.29) and the positive association in premenopausal women was attenuated after exclusion of women with anovulatory cycles.

**Table 4:** Doubling of sex steroids and SHBG and breast cancer risk: Nurses' Health Study II, 1999 to 2009

	All Cases*		Premenopausal at Diagnosis		Postmenopausal at Diagnosis		*P*_het**_
			
	cases/		cases/		cases/		
	controls	OR (95% CI)	controls	OR (95% CI)	controls	OR (95% CI)	
**FOLLICULAR**							
**Estradiol**							
All cancers	462/909	1.0 (0.9 to 1.2)	340/677	1.1 (0.9 to 1.3)	92/176	0.6 (0.4 to 0.9)	< 0.01
Invasive	299/909	1.0 (0.9 to 1.2)	221/677	1.2 (1.0 to 1.4)	62/176	0.6 (0.4 to 0.9)	< 0.01
ER+/PR+	201/909	1.0 (0.8 to 1.2)	152/677	1.2 (0.9 to 1.5)	43/176	0.6 (0.4 to 0.9)	0.01
**Free Estradiol**							
All cancers	447/886	1.0 (0.8 to 1.1)	330/668	1.1 (0.9 to 1.4)	86/162	0.5 (0.4 to 0.8)	< 0.01
Invasive	288/886	0.9 (0.8 to 1.1)	214/668	1.2 (0.9 to 1.5)	57/162	0.5 (0.3 to 0.7)	< 0.01
ER+/PR+	194/886	0.9 (0.8 to 1.1)	146/668	1.2 (0.9 to 1.5)	41/162	0.4 (0.3 to 0.7)	< 0.01
**Estrone**							
All cancers	469/920	1.0 (0.8 to 1.3)	347/692	1.0 (0.8 to 1.3)	91/173	1.0 (0.6 to 1.7)	0.83
Invasive	305/920	1.0 (0.8 to 1.2)	227/692	0.9 (0.7 to 1.3)	61/173	0.8 (0.5 to 1.4)	0.85
ER+/PR+	206/920	1.2 (0.9 to 1.5)	156/692	1.1 (0.8 to 1.6)	43/173	1.0 (0.6 to 1.8)	0.95
**LUTEAL**							
**Estradiol**							
All cancers	479/959	1.0 (0.8 to 1.2)	347/710	1.0 (0.8 to 1.3)	98/190	1.1 (0.7 to 1.7)	0.82
Invasive	316/959	1.2 (1.0 to 1.4)	232/710	1.2 (1.0 to 1.6)	67/190	1.1 (0.7 to 1.7)	0.66
ER+/PR+	215/959	1.2 (1.0 to 1.5)	162/710	1.3 (1.0 to 1.8)	47/190	1.2 (0.8 to 2.1)	0.80
**Free estradiol**							
All cancers	469/944	1.0 (0.8 to 1.2)	339/702	1.0 (0.8 to 1.3)	96/183	1.1 (0.7 to 1.7)	0.61
Invasive	309/944	1.1 (0.9 to 1.4)	227/702	1.2 (0.9 to 1.5)	65/183	1.0 (0.7 to 1.7)	0.52
ER+/PR+	209/944	1.2 (0.9 to 1.5)	157/702	1.2 (0.9 to 1.7)	46/183	1.3 (0.8 to 2.3)	0.88
**Estrone**							
All cancers	500/1005	1.0 (0.8 to 1.2)	366/751	0.9 (0.7 to 1.2)	99/194	1.3 (0.8 to 2.1)	0.22
Invasive	328/1005	1.1 (0.9 to 1.4)	242/751	1.0 (0.8 to 1.3)	67/194	1.4 (0.8 to 2.2)	0.34
ER+/PR+	221/1005	1.1 (0.8 to 1.4)	167/751	1.0 (0.7 to 1.4)	47/194	1.5 (0.9 to 2.7)	0.26
**Progesterone**							
All cancers	501/1009	1.0 (0.9 to 1.1)	368/753	1.0 (0.9 to 1.2)	96/193	0.9 (0.7 to 1.1)	0.24
Invasive	329/1009	1.0 (0.9 to 1.1)	243/753	1.1 (0.9 to 1.2)	66/193	0.8 (0.7 to 1.0)	0.29
ER+/PR+	222/1009	1.0 (0.9 to 1.1)	168/753	1.0 (0.9 to 1.2)	46/193	0.8 (0.6 to 1.0)	0.42
**LUTEAL AND UNTIMED**							
**Testosterone**							
All cancers	623/1245	1.2 (1.0 to 1.4)	417/858	1.1 (0.9 to 1.4)	141/271	1.3 (0.8 to 2.0)	0.52
Invasive	421/1245	1.2 (1.0 to 1.5)	278/858	1.1 (0.8 to 1.4)	100/271	1.8 (1.1 to 2.8)	0.16
ER+/PR+	290/1245	1.3 (1.0 to 1.7)	192/858	1.2 (0.9 to 1.7)	70/271	1.9 (1.1 to 3.4)	0.41
**Free testosterone**							
All cancers	617/1237	1.0 (0.9 to 1.1)	414/853	1.0 (0.8 to 1.2)	138/267	1.2 (0.8 to 1.8)	0.23
Invasive	416/1237	1.0 (0.8 to 1.2)	276/853	0.9 (0.7 to 1.1)	97/267	1.3 (0.9 to 1.9)	0.24
ER+/PR+	287/1237	1.1 (0.9 to 1.3)	190/853	1.0 (0.8 to 1.3)	69/267	1.7 (1.0 to 2.6)	0.22
**DHEAS**							
All cancers	628/1254	1.1 (1.0 to 1.2)	422/861	1.1 (0.9 to 1.2)	141/276	1.1 (0.8 to 1.4)	0.71
Invasive	423/1254	1.1 (0.9 to 1.2)	280/861	1.0 (0.9 to 1.2)	100/276	1.1 (0.8 to 1.5)	0.38
ER+/PR+	291/1254	1.2 (1.0 to 1.4)	193/861	1.1 (0.9 to 1.4)	70/276	1.3 (0.9 to 1.9)	0.22
**SHBG**							
All cancers	624/1246	1.1 (1.0 to 1.3)	421/859	1.2 (1.0 to 1.4)	138/270	0.9 (0.7 to 1.4)	0.27
Invasive	420/1246	1.2 (1.0 to 1.4)	280/859	1.2 (1.0 to 1.5)	97/270	1.0 (0.7 to 1.5)	0.66
ER+/PR+	290/1246	1.1 (0.9 to 1.3)	193/859	1.2 (0.9 to 1.5)	69/270	0.9 (0.6 to 1.3)	0.37

*Includes women of all menopausal statuses at diagnosis (premenopausal, postmenopausal and unknown). **P_het _represents heterogeneity between premenopausal vs. postmenopausal status at diagnosis, among women premenopausal at blood collection. All cancers: Conditional logistic regression for all cancers controlling for age at menarche, parity/age at first birth, BMI at age 18, family history of breast cancer, history of benign breast disease. Invasive, ER+/PR+ tumors: Unconditional logistic regression for invasive and ER+/PR+ disease controlling for factors listed above and matching factors.

The associations between premenopausal androgens and breast cancer generally appeared stronger among women who were postmenopausal at diagnosis, but these differences by menopausal status were not statistically significant (*P*_het _> 0.05). For example, for invasive breast cancer, premenopausal testosterone was not associated with premenopausal disease (OR: 1.1, 95% CI: 0.8 to 1.4), but was significantly associated with postmenopausal disease (OR: 1.8, 95% CI: 1.1 to 2.8) with a similar pattern for ER+/PR+ cancers, as well as for free testosterone.

Results were similar when we excluded cases diagnosed within two years of blood draw. A small number of women providing untimed samples reported exogenous hormone use at blood collection (*n *= 24 cases, 36 controls). Given the positive association between exogenous hormone use and SHBG in this population (*P *< 0.001) we conducted sub-analyses for SHBG and free testosterone excluding these women; results were similar to the overall results. Further, with only a few exceptions, the associations did not vary significantly by BMI at blood collection, duration of past oral contraceptive (OC) use, menstrual cycle characteristics (that is, length, regularity), or age at blood draw. For follicular free estradiol, no significant association was observed with BMI < 25 (doubling OR: 1.0, 95% CI: 0.8 to 1.3) and an inverse association was observed for women with BMI ≥ 25 (doubling OR: 0.6, 95% CI: 0.4 to 0.9; *P*_het _= 0.04). Similarly, there was no association between progesterone and breast cancer risk in women age < 45 at blood draw (doubling ER+/PR+, OR: 1.1, 95% CI: 0.9 to 1.3) and a suggestively inverse association in women ≥ 45 (doubling ER+/PR+, OR: 0.8, 95% CI: 0.7 to 1.0).

Estradiol, testosterone and DHEAS appeared more strongly associated with ER+/PR+ tumors, as compared to overall associations (Tables 2, 3, 4). However, with one exception we observed no statistical heterogeneity by tumor hormone receptor subtypes (ER+/PR+ vs. ER-/PR-, all *P*_het _> 0.05). For follicular estrone, there was a suggestive positive association for ER+/PR+ disease (doubling OR: 1.2, 95% CI: 0.9 to 1.7) and an inverse association for ER-/PR- disease (doubling OR: 0.6, 95% CI: 0.3 to 1.3) (*P*_het _= 0.01), although these results were limited by a small number of ER-/PR- cases (60 timed samples).

We conducted analyses cross-classifying follicular and luteal phase estradiol and no clear pattern emerged. For example, for invasive breast cancer ORs were 1.7 (95% CI: 0.8 to 1.8) for high follicular/low luteal estradiol, 1.5 (95% CI: 1.0 to 2.3) for low follicular/high luteal estradiol, and 1.3 (95% CI: 0.9 to 1.9) for high estradiol in both phases, all relative to low estradiol in both phases.

We corrected the hormone/breast cancer associations for within-person variability and laboratory measurement error using our prior reproducibility study results [13,20]. Results for hormones with either high intraclass correlation coefficients (ICCs) or weak (or null) associations were largely similar before and after correction. For example, a doubling of DHEAS (ICC = 0.87) was associated with a 10% increase in risk of invasive disease both before and after measurement error correction (before, OR: 1.1, 95% CI: 0.9 to 1.2); after, OR: 1.1, 95% CI: 0.9 to 1.3). Results were substantially strengthened for several of the hormones, such as follicular estradiol and testosterone. A doubling of follicular estradiol (ICC = 0.45) was associated with a 20% increase in risk of invasive premenopausal breast cancer in uncorrected estimates (OR: 1.2, 95% CI: (1.0 to 1.4)), but a 50% increase in risk in corrected estimates (OR: 1.5, 95% CI: 0.9 to 2.4), though measurement error adjusted results were no longer statistically significant. Similarly, a doubling of testosterone (ICC = 0.69) was associated with an 80% increase in risk of invasive postmenopausal breast cancer (OR: 1.8, 95% CI: 1.1 to 2.8) before measurement error correction, and a 2.5-fold increase in risk after correction (OR = 2.5, 95% CI: 1.2 to 5.4).

## Discussion

In this prospective study of premenopausal plasma sex steroids and SHBG and breast cancer risk, we found positive associations between androgens and risk of both invasive and ER+/PR+ breast cancer, as well as luteal estradiol and risk of ER+/PR+ tumors. Several of the associations varied by menopausal status at breast cancer diagnosis. Total and free estradiol levels in the follicular phase were positively, but not significantly, associated with premenopausal invasive and ER+/PR+ disease but significantly inversely associated with postmenopausal disease. Testosterone levels also appeared more strongly associated with postmenopausal breast cancer, although these differences were not statistically different. We observed no significant associations when evaluating all cancers combined.

The positive associations between endogenous estrogens and androgens and postmenopausal breast cancer risk are well established in prior epidemiologic studies [4,31-33], and biologic mechanisms are well described for estrogens [1,34]. Estrogens are associated with increased proliferation and decreased apoptosis, and may promote proliferation of cells with genetic mutations [1]. The biologic mechanism between androgens and breast cancer is less established, with androgens demonstrating both growth inhibitory [2,3] and proliferative [3] effects in breast cancer cell lines. In animal models, androgens have been shown to inhibit proliferation [35-37] and the androgen receptor antagonist flutamide has been shown to increase epithelial cell proliferation [35]. Breast tissue can convert androgens to estrogens via aromatase [38] and, therefore, androgens, through their conversion to estrogen, also may be indirectly associated with breast cancer risk.

There is an inherent complexity in measuring estrogens in premenopausal women given the variation in estrogen levels across the menstrual cycle, and prior studies have accounted for this in different ways. The eight prior prospective studies [5-7,9-13] either did not account for menstrual cycle day [11], matched cases and controls on cycle day [5-7,10], matched cases and controls on cycle day and used a spline regression model to adjust for cycle differences [9,12], or, as in our prior analysis, restricted sample collection to specific windows in the menstrual cycle and matched on cycle day [13]. Six of the eight prior studies [5-7,9-11] had fewer than 100 cases, with four of the six [5,6,9,11] finding non-significant or suggestive positive associations. Kaaks *et al. *[12], in the largest prior study (case *n *= 285) in the European Prospective Investigation into Cancer and Nutrition (EPIC) cohort, found no association with invasive breast cancer risk for either estrone (4^th ^vs. 1^st ^quartile OR: 1.2, 95%: 0.7 to 1.9, *P*_trend _= 0.46) or estradiol (OR: 1.0, 95%: 0.7 to 1.5, *P*_trend _= 0.89). A prior analysis in a subset of the population included in the current analysis [13], in women predominantly premenopausal at diagnosis (*n *= 197 cases; *n *= 192 premenopausal at diagnosis), found a significant positive association between follicular estradiol with breast cancer overall (4^th ^vs. 1^st ^quartile OR: 2.1, 95% CI: 1.1 to 4.1, *P*_trend _= 0.08), that appeared somewhat stronger for invasive and ER+/PR+ disease. Results in the present study, with the addition of 317 timed cases, are attenuated relative to our prior findings, though we continue to see the suggestion of a weak positive association for follicular estradiol and invasive breast cancer diagnosed in premenopausal women. In our prior analysis, we saw no clear association between luteal estradiol and breast cancer; results in the current analysis are suggestive of an association between luteal estradiol and invasive and ER+/PR+ tumors.

The association between circulating premenopausal androgens and breast cancer risk has been investigated in seven prospective studies [5,6,8,10-12,15], in addition to the NHSII [13,14]. In the EPIC cohort (*n *= 370 cases), significant positive associations with breast cancer risk were observed for both testosterone (4^th ^vs. 1^st ^quartile OR: 1.7, 95% CI: 1.2 to 2.6, *P*_trend _= 0.01) and DHEAS (OR = 1.5, 95% CI: 1.0 to 2.1, *P*_trend _= 0.10) [12]. Recent results from the New York University Women's Health Study (NYUWHS) (*n *= 356 cases) [15], are in agreement, where an approximately two-fold increase in risk was observed in the highest quintile of both testosterone (OR: 2.2, 95% CI: 1.3 to 3.5, *P*_trend _= 0.03) and free testosterone (OR: 1.9, 95% CI: 1.2 to 2.9, *P*_trend _= 0.01), though a weaker, non-significant positive association was observed with DHEAS (OR: 1.3, 95% CI: 0.8 to 2.1, *P*_trend _= 0.20). In our initial report in the NHSII, positive associations were seen for luteal testosterone (OR: 1.6, 95% CI: 0.9 to 2.8, *P*_trend _= 0.10), free testosterone (OR: 1.4, 95% CI: 0.8 to 2.5, *P*_trend _= 0.14) [13], and DHEAS (OR: 1.3, 95% CI: 0.9 to 2.1, *P*_trend _= 0.08) [14]. In the current much larger study, our findings are somewhat weaker, although a number of associations remained statistically significant. Thus, overall, data from prospective studies are quite consistent in showing that premenopausal androgen levels predict later breast cancer risk.

In this updated analysis, with six more years of follow-up, we observed an attenuation of our previously published results, particularly for follicular estradiol. Cases included in the previous analysis were similar to the new cases with regards to case characteristics (for example, invasive vs. *in situ*, hormone receptor status, grade, stage), and risk factor status (for example, family history of breast cancer). Data from our prior reproducibility study [20] suggest androgens and SHBG are quite stable over at least three years (ICC range: 0.73 (testosterone) to 0.89 (SHBG)) and do not change substantially at menopause while ICCs for the estrogens are lower (ICC range: 0.33 (estrone) to 0.45 (estradiol)), and levels change markedly around menopause. It is possible, particularly for the estrogens, that steroid hormones measured closer to diagnosis are a stronger predictor of disease, or are most relevant to risk as promoters of early stage tumorigenesis.

There are little prior data to suggest a differential effect of premenopausal sex steroids in relation to pre- vs. postmenopausal disease. In the current analysis, follicular estradiol was associated with a suggestively increased risk of premenopausal breast cancer and significantly inverse risk of postmenopausal disease. Given this novel finding, and the considerably different hormonal milieu in pre- vs. postmenopausal women, further investigation is warranted. Although speculative, because estradiol levels are substantially lower after menopause, women with higher premenopausal follicular estradiol may experience the greatest change in estradiol levels from pre- to post menopause, which may in turn confer a lower risk, at least over the short term. There is some indirect evidence to support a role for changes in endogenous estrogen levels in breast cancer etiology. For example, discontinuation of exogenous postmenopausal hormones (PMH) is associated with reduced proliferation in ER+ tumors [39] and postmenopausal weight loss, which is associated with a decrease in estrogen levels, is associated with lower breast cancer risk [40,41].

Our androgen/breast cancer associations appeared stronger in women postmenopausal at diagnosis, although the differences by menopausal status were not statistically significant. This has been evaluated in few other cohorts. In the NYUWHS, the association between testosterone and breast cancer risk was suggestively stronger in women postmenopausal at diagnosis (for doubling of testosterone: premenopausal, OR: 1.4, 95% CI: 0.8 to 2.3, *P*_trend _= 0.23; postmenopausal, OR: 1.7, 95% CI: 1.1 to 2.6, *P*_trend _= 0.02), although this difference also was not statistically significant (*P *for interaction > 0.15) [15]. Similarly, in the Columbia, Missouri cohort, results for total testosterone and bioavailable testosterone were stronger among women ages 55 and older at diagnosis (for example, 4^th ^vs. 1^st ^quartile, overall results for testosterone, OR: 3.3, 95% CI: 1.5 to 7.5, *P*_trend _= 0.006; among women ≥ 55 at diagnosis, OR: 4.5, 95% CI: 1.6 to 13.0, *P*_trend _= 0.009) [5]. However, in contrast to these findings, in the EPIC study associations were somewhat stronger for both testosterone and DHEAS in women ages ≤ 49 years at diagnosis [12]. Clearly, additional studies are necessary to address this potential heterogeneity. The influence of androgens on breast tissue in a high estrogen environment may differ from those in a low estrogen environment though this hypothesis has not been adequately explored. Androgen levels do not change substantially at menopause [42,43], and prior prospective studies consistently link postmenopausal androgens to breast cancer risk [4]. Therefore, it is possible that premenopausal androgen levels are a marker for postmenopausal levels, which are then determinants of postmenopausal breast cancer risk.

Among prior prospective assessments of premenopausal progesterone levels and breast cancer risk, three [6,8,10] of four small studies (case n < 100) found non-significant inverse associations, and the larger EPIC cohort reported a significant inverse association [12]. In our prior analyses in the NHSII, we observed no association between progesterone and breast cancer risk [13], and this was confirmed in the current expanded analysis. The ICC over three years for progesterone is 0.29, evidence that it is not well measured with one sample. Prior prospective studies [5-8,12,13,15] did not find a statistically significant association between premenopausal SHBG and breast cancer risk, in agreement with our findings.

Our study has both strengths and limitations. This is the largest prospective study to date, although we had limited power in analyses stratified by menopausal status at diagnosis (particularly postmenopausal cases). Our samples were carefully timed in the menstrual cycle. We used highly specific and sensitive assays, and laboratory CVs were excellent. Additionally, we are among the first to present data for premenopausal hormones and breast cancer by tumor hormone receptor status. Although we are limited to samples collected during one menstrual cycle, a prior reproducibility study provides evidence of reasonable stability across a three-year period (follicular estradiol: 0.38 to DHEAS: 0.86), except for luteal progesterone (ICC = 0.29) [20]. We also used these reproducibility data to correct for measurement error and showed that several of the associations may be substantially stronger than observed.

## Conclusions

In summary, this large, prospective study of circulating premenopausal hormones and breast cancer risk provides some evidence for moderate associations between plasma hormones and breast cancer risk. We provide suggestive evidence of a role for premenopausal estrogens and androgens in postmenopausal disease, with premenopausal estrogens inversely and premenopausal androgens positively associated with postmenopausal breast cancer. However, further work is needed to explore the relationships between premenopausal hormone levels and postmenopausal disease.

## Abbreviations

BMI: body mass index; CI: confidence interval; CVs: coefficients of variation; DHEAS: dehydroepiandrosterone sulfate; EPIC: European Prospective Investigation into Cancer and Nutrition cohort; ER+: estrogen receptor positive; ICC: intraclass correlation coefficients; NHSII: Nurses' Health Study II; NYUWHS: New York University Women's Health Study; OR: odds ratio; PMH: postmenopausal hormones; PR+: progesterone receptor positive; RR: relative risk; SHBG: sex hormone binding globulin

## Competing interests

The authors state that they have no competing interests.

## Authors' contributions

RTF analyzed and interpreted the data and wrote the manuscript. AHE conceived of the study design, acquired the data, contributed to the interpretation and analysis of data, and critically reviewed the manuscript. DS provided statistical expertise, contributed to the interpretation and analysis of data, and critically reviewed the manuscript. RLB contributed to the interpretation of data and critically reviewed the manuscript. WCW and SEH conceived of the study design, secured funding, acquired the data, contributed to the interpretation and analysis of data, and critically reviewed the manuscript. All authors read and approved the final manuscript.

## Supplementary Material

Additional file 1 **Table S1**. Quartiles of premenopausal sex steroids and SHBG and breast cancer risk, by menopausal status at diagnosis: Nurses' Health Study II, 1999 to 2009. Click here for file
