# Supplementary material for: Premenopausal endogenous steroid hormones and breast cancer risk: results from the Nurses' Health Study II
Source: Breast Cancer Res. 2013 Mar 6;15(2):R19. doi: 10.1186/bcr3394 (PMC3672790; doi:10.1186/bcr3394)
Supplement: Additional file 1 — Table S1. Quartiles of premenopausal sex steroids and SHBG and breast cancer risk, by menopausal status at diagnosis: Nurses' Health Study II, 1999 to 2009. [file bcr3394-S1.DOC]

| **Supplemental Table. Quartiles of premenopausal sex steroids and SHBG and breast cancer risk, by menopausal status at diagnosis: Nurses' Health Study II, 1999-2009** | | | | | | | | | | | | | | | | | | | | | | | | | |  | | | |
| --- | --- | --- | --- | --- | --- | --- | --- | --- | --- | --- | --- | --- | --- | --- | --- | --- | --- | --- | --- | --- | --- | --- | --- | --- | --- | --- | --- | --- | --- |
|  |  |  |  | | **1st Quartile** |  | | **2nd Quartile** | |  | | **3rd Quartile** |  | | | **4th Quartile** | | |  | | |  | | | | | |  | |
|  |  |  |  | | **OR (95% CI)** |  | | **OR (95% CI)** | |  | | **OR (95% CI)** |  | | | **OR (95% CI)** | | |  | | | **p_trend_** | | | | | |  | |
| **PREMENOPAUSAL AT DIAGNOSIS** | | | | | | | | | |  |  | | |  | | |  | | | |  | | |  | | | | |  |
| **FOLLICULAR** | |  |  | |  |  | |  | |  | |  |  | | |  | | |  | | |  | | | | | |  | |
| **Estradiol** | |  |  | |  |  | |  | |  | |  |  | | |  | | |  | | |  | | | | | |  | |
|  | Cutpoints, pg/mL* |  |  | | <32 |  | | ≥32-45.9 | |  | | ≥46-65.9 |  | | | ≥66 | | |  | | |  | | | | | |  | |
|  | No. cases/No. controls |  |  | | 69/153 |  | | 85/178 | |  | | 96/166 |  | | | 90/180 | | |  | | |  | | | | | |  | |
|  | all cancers |  |  | | 1.0 (referent) |  | | 1.2 (0.8-1.7) | |  | | 1.2 (0.8-1.7) |  | | | 1.1 (0.7-1.7) | | |  | | | 0.68 | | | | | |  | |
|  | invasive |  |  | | 1.0 (referent) |  | | 1.2 (0.8-2.0) | |  | | 1.4 (0.9-2.3) |  | | | 1.3 (0.8-2.1) | | |  | | | 0.28 | | | | | |  | |
|  | ER+/PR+ |  |  | | 1.0 (referent) |  | | 1.1 (0.6-1.9) | |  | | 1.5 (0.9-2.6) |  | | | 1.2 (0.7-2.1) | | |  | | | 0.45 | | | | | |  | |
|  |  |  |  | |  |  | |  | |  | |  |  | | |  | | |  | | |  | | | | | |  | |
| **Free Estradiol** | |  |  | |  |  | |  | |  | |  |  | | |  | | |  | | |  | | | | | |  | |
|  | Cutpoints, pg/mL |  |  | | <0.43 |  | | ≥0.43-0.579 | |  | | ≥0.58-0.819 |  | | | ≥0.82 | | |  | | |  | | | | | |  | |
|  | No. cases/No. controls |  |  | | 64/159 |  | | 88/167 | |  | | 97/181 |  | | | 81/161 | | |  | | |  | | | | | |  | |
|  | all cancers |  |  | | 1.0 (referent) |  | | 1.3 (0.8-1.9) | |  | | 1.2 (0.8-1.8) |  | | | 1.3 (0.8-2.0) | | |  | | | 0.42 | | | | | |  | |
|  | invasive |  |  | | 1.0 (referent) |  | | 1.4 (0.9-2.3) | |  | | 1.5 (1.0-2.5) |  | | | 1.4 (0.9-2.3) | | |  | | | 0.19 | | | | | |  | |
|  | ER+/PR+ |  |  | | 1.0 (referent) |  | | 1.6 (0.9-2.8) | |  | | 1.8 (1.0-3.1) |  | | | 1.4 (0.8-2.6) | | |  | | | 0.28 | | | | | |  | |
|  |  |  |  | |  |  | |  | |  | |  |  | | |  | | |  | | |  | | | | | |  | |
| **Estrone** | |  |  | |  |  | |  | |  | |  |  | | |  | | |  | | |  | | | | | |  | |
|  | Cutpoints, pg/mL |  |  | | <32 |  | | ≥32-40.9 | |  | | ≥41-52.9 |  | | | ≥53 | | |  | | |  | | | | | |  | |
|  | No. cases/No. controls |  |  | | 73/157 |  | | 88/177 | |  | | 104/184 |  | | | 82/174 | | |  | | |  | | | | | |  | |
|  | all cancers |  |  | | 1.0 (referent) |  | | 1.0 (0.7-1.5) | |  | | 1.2 (0.8-1.7) |  | | | 1.0 (0.7-1.5) | | |  | | | 0.72 | | | | | |  | |
|  | invasive |  |  | | 1.0 (referent) |  | | 1.1 (0.7-1.7) | |  | | 1.1 (0.7-1.6) |  | | | 1.0 (0.6-1.5) | | |  | | | 0.71 | | | | | |  | |
|  | ER+/PR+ |  |  | | 1.0 (referent) |  | | 1.3 (0.8-2.2) | |  | | 1.3 (0.8-2.3) |  | | | 1.2 (0.7-2.0) | | |  | | | 0.76 | | | | | |  | |
|  |  |  |  | |  |  | |  | |  | |  |  | | |  | | |  | | |  | | | | | |  | |
| **LUTEAL** | |  |  | |  |  | |  | |  | |  |  | | |  | | |  | | |  | | | | | |  | |
| **Estradiol** | |  |  | |  |  | |  | |  | |  |  | | |  | | |  | | |  | | | | | |  | |
|  | Cutpoints, pg/mL |  |  | | <98 |  | | ≥98-130.9 | |  | | ≥131-172.9 |  | | | ≥173 | | |  | | |  | | | | | |  | |
|  | No. cases/No. controls |  |  | | 68/174 |  | | 99/181 | |  | | 113/178 |  | | | 67/177 | | |  | | |  | | | | | |  | |
|  | all cancers |  |  | | 1.0 (referent) |  | | 1.3 (0.9-2.0) | |  | | 1.7 (1.1-2.5) |  | | | 0.9 (0.6-1.5) | | |  | | | 0.92 | | | | | |  | |
|  | invasive |  |  | | 1.0 (referent) |  | | 1.5 (0.9-2.4) | |  | | 2.0 (1.1-3.1) |  | | | 1.3 (0.8-2.1) | | |  | | | 0.18 | | | | | |  | |
|  | ER+/PR+ |  |  | | 1.0 (referent) |  | | 1.6 (0.9-2.7) | |  | | 2.3 (1.4-4.0) |  | | | 1.5 (0.9-2.7) | | |  | | | 0.07 | | | | | |  | |
| ***Anovulatory Excluded*** | |  |  | |  |  | |  | |  | |  |  | | |  | | |  | | |  | | | | | |  | |
|  | all cancers |  |  | | 1.0 (referent) |  | | 1.0 (0.7-1.6) | |  | | 1.3 (0.9-2.1) |  | | | 0.8 (0.5-1.3) | | |  | | | 0.61 | | | | | |  | |
|  | invasive |  |  | | 1.0 (referent) |  | | 1.2 (0.8-2.0) | |  | | 1.5 (0.9-2.4) |  | | | 1.0 (0.6-1.6) | | |  | | | 0.88 | | | | | |  | |
|  | ER+/PR+ |  |  | | 1.0 (referent) |  | | 1.3 (0.7-2.3) | |  | | 1.7 (1.0-3.0) |  | | | 1.2 (0.7-2.2) | | |  | | | 0.40 | | | | | |  | |
|  |  |  |  | |  |  | |  | |  | |  |  | | |  | | |  | | |  | | | | | |  | |
| **Free Estradiol** | |  |  | |  |  | |  | |  | |  |  | | |  | | |  | | |  | | | | | |  | |
|  | Cutpoints, pg/mL |  |  | | <1.26 |  | | ≥1.26-1.689 | |  | | ≥1.69-2.219 |  | | | ≥2.22 | | |  | | |  | | | | | |  | |
|  | No. cases/No. controls |  |  | | 75/172 |  | | 104/176 | |  | | 84/183 |  | | | 76/171 | | |  | | |  | | | | | |  | |
|  | all cancers |  |  | | 1.0 (referent) |  | | 1.6 (1.1-2.4) | |  | | 1.2 (0.8-1.8) |  | | | 1.1 (0.7-1.7) | | |  | | | 0.92 | | | | | |  | |
|  | invasive |  |  | | 1.0 (referent) |  | | 1.5 (1.0-2.4) | |  | | 1.3 (0.8-2.0) |  | | | 1.3 (0.8-2.1) | | |  | | | 0.39 | | | | | |  | |
|  | ER+/PR+ |  |  | | 1.0 (referent) |  | | 1.6 (0.9-2.7) | |  | | 1.3 (0.8-2.3) |  | | | 1.5 (0.9-2.6) | | |  | | | 0.24 | | | | | |  | |
| ***Anovulatory Excluded*** | |  |  | |  |  | |  | |  | |  |  | | |  | | |  | | |  | | | | | |  | |
|  | all cancers |  |  | | 1.0 (referent) |  | | 1.5 (1.0-2.3) | |  | | 1.2 (0.7-1.8) |  | | | 0.9 (0.6-1.5) | | |  | | | 0.53 | | | | | |  | |
|  | invasive |  |  | | 1.0 (referent) |  | | 1.3 (0.8-2.1) | |  | | 1.2 (0.7-1.9) |  | | | 0.9 (0.6-1.5) | | |  | | | 0.66 | | | | | |  | |
|  | ER+/PR+ |  |  | | 1.0 (referent) |  | | 1.4 (0.8-2.4) | |  | | 1.2 (0.7-2.1) |  | | | 1.1 (0.6-2.0) | | |  | | | 0.91 | | | | | |  | |
|  |  |  |  | |  |  | |  | |  | |  |  | | |  | | |  | | |  | | | | | |  | |
| **Estrone** | |  |  | |  |  | |  | |  | |  |  | | |  | | |  | | |  | | | | | |  | |
|  | Cutpoints, pg/mL |  |  | | <65 |  | | ≥65-82.9 | |  | | ≥83-105.9 |  | | | ≥106 | | |  | | |  | | | | | |  | |
|  | No. cases/No. controls |  |  | | 100/179 |  | | 94/187 | |  | | 78/201 |  | | | 94/184 | | |  | | |  | | | | | |  | |
|  | all cancers |  |  | | 1.0 (referent) |  | | 0.9 (0.6-1.3) | |  | | 0.7 (0.5-1.1) |  | | | 0.9 (0.6-1.3) | | |  | | | 0.38 | | | | | |  | |
|  | invasive |  |  | | 1.0 (referent) |  | | 1.0 (0.6-1.5) | |  | | 0.6 (0.4-0.9) |  | | | 1.0 (0.7-1.5) | | |  | | | 0.50 | | | | | |  | |
|  | ER+/PR+ |  |  | | 1.0 (referent) |  | | 0.9 (0.5-1.4) | |  | | 0.7 (0.4-1.2) |  | | | 0.9 (0.5-1.4) | | |  | | | 0.46 | | | | | |  | |
| ***Anovulatory Excluded*** | |  |  | |  |  | |  | |  | |  |  | | |  | | |  | | |  | | | | | |  | |
|  | all cancers |  |  | | 1.0 (referent) |  | | 0.9 (0.6-1.3) | |  | | 0.7 (0.5-1.1) |  | | | 0.7 (0.5-1.1) | | |  | | | 0.12 | | | | | |  | |
|  | invasive |  |  | | 1.0 (referent) |  | | 0.9 (0.6-1.4) | |  | | 0.6 (0.4-0.9) |  | | | 0.8 (0.5-1.2) | | |  | | | 0.12 | | | | | |  | |
|  | ER+/PR+ |  |  | | 1.0 (referent) |  | | 0.8 (0.5-1.4) | |  | | 0.7 (0.4-1.2) |  | | | 0.7 (0.4-1.2) | | |  | | | 0.18 | | | | | |  | |
|  |  |  |  | |  |  | |  | |  | |  |  | | |  | | |  | | |  | | | | | |  | |
| **Progesterone** | |  |  | |  |  | |  | |  | |  |  | | |  | | |  | | |  | | | | | |  | |
|  | Cutpoints, ng/dL |  |  | | <888 |  | | ≥888-1409.9 | |  | | ≥1410-2004.9 |  | | | ≥2005 | | |  | | |  | | | | | |  | |
|  | No. cases/No. controls |  |  | | 81/175 |  | | 84/199 | |  | | 101/200 |  | | | 102/179 | | |  | | |  | | | | | |  | |
|  | all cancers |  |  | | 1.0 (referent) |  | | 1.0 (0.6-1.4) | |  | | 1.1 (0.8-1.7) |  | | | 1.3 (0.9-2.0) | | |  | | | 0.21 | | | | | |  | |
|  | invasive |  |  | | 1.0 (referent) |  | | 0.9 (0.5-1.4) | |  | | 1.2 (0.8-1.8) |  | | | 1.2 (0.8-1.9) | | |  | | | 0.28 | | | | | |  | |
|  | ER+/PR+ |  |  | | 1.0 (referent) |  | | 0.7 (0.4-1.2) | |  | | 1.0 (0.6-1.7) |  | | | 1.2 (0.7-1.9) | | |  | | | 0.55 | | | | | |  | |
| ***Anovulatory Excluded*** | | | |  | | |  | |  |  | |  | | |  | | |  | | | | |  | |  | | | | |
|  | all cancers |  |  | | 1.0 (referent) |  | | 0.9 (0.5-1.4) | |  | | 1.1 (0.7-1.8) |  | | | 1.2 (0.8-2.1) | | |  | | | 0.27 | | | | | |  | |
|  | invasive |  |  | | 1.0 (referent) |  | | 0.7 (0.4-1.2) | |  | | 1.0 (0.6-1.7) |  | | | 1.1 (0.6-1.8) | | |  | | | 0.56 | | | | | |  | |
|  | ER+/PR+ |  |  | | 1.0 (referent) |  | | 0.6 (0.3-1.1) | |  | | 0.8 (0.5-1.5) |  | | | 1.0 (0.5-1.7) | | |  | | | 0.91 | | | | | |  | |
|  |  |  |  | |  |  | |  | |  | |  |  | | |  | | |  | | |  | | | | | |  | |
| **LUTEAL AND UNTIMED** | |  |  | |  |  | |  | |  | |  |  | | |  | | |  | | |  | | | | | |  | |
| **Testosterone** | |  |  | |  |  | |  | |  | |  |  | | |  | | |  | | |  | | | | | |  | |
|  | Cutpoints, ng/dL |  |  | | <20 |  | | ≥20-24.9 | |  | | ≥25-32.9 |  | | | ≥33 | | |  | | |  | | | | | |  | |
|  | No. cases/No. controls |  |  | | 100/217 |  | | 89/205 | |  | | 107/210 |  | | | 121/226 | | |  | | |  | | | | | |  | |
|  | all cancers |  |  | | 1.0 (referent) |  | | 0.9 (0.6-1.3) | |  | | 1.0 (0.7-1.4) |  | | | 1.2 (0.8-1.7) | | |  | | | 0.36 | | | | | |  | |
|  | invasive |  |  | | 1.0 (referent) |  | | 1.0 (0.7-1.5) | |  | | 0.9 (0.6-1.4) |  | | | 1.1 (0.7-1.6) | | |  | | | 0.69 | | | | | |  | |
|  | ER+/PR+ |  |  | | 1.0 (referent) |  | | 1.3 (0.8-2.0) | |  | | 1.0 (0.6-1.6) |  | | | 1.4 (0.9-2.2) | | |  | | | 0.25 | | | | | |  | |
|  |  |  |  | |  |  | |  | |  | |  |  | | |  | | |  | | |  | | | | | |  | |
| **Free Testosterone** | |  |  | |  |  | |  | |  | |  |  | | |  | | |  | | |  | | | | | |  | |
|  | Cutpoints, ng/dL |  |  | | <0.13 |  | | ≥0.13-0.189 | |  | | ≥0.19-0.279 |  | | | ≥0.28 | | |  | | |  | | | | | |  | |
|  | No. cases/No. controls |  |  | | 111/215 |  | | 94/223 | |  | | 102/189 |  | | | 107/226 | | |  | | |  | | | | | |  | |
|  | all cancers |  |  | | 1.0 (referent) |  | | 0.8 (0.6-1.1) | |  | | 1.0 (0.7-1.5) |  | | | 1.0 (0.7-1.5) | | |  | | | 0.77 | | | | | |  | |
|  | invasive |  |  | | 1.0 (referent) |  | | 0.9 (0.6-1.3) | |  | | 1.2 (0.8-1.8) |  | | | 0.8 (0.6-1.3) | | |  | | | 0.72 | | | | | |  | |
|  | ER+/PR+ |  |  | | 1.0 (referent) |  | | 0.9 (0.5-1.4) | |  | | 1.2 (0.8-1.9) |  | | | 1.0 (0.6-1.6) | | |  | | | 0.68 | | | | | |  | |
|  |  |  |  | |  |  | |  | |  | |  |  | | |  | | |  | | |  | | | | | |  | |
| **DHEAS** | |  |  | |  |  | |  | |  | |  |  | | |  | | |  | | |  | | | | | |  | |
|  | Cutpoints, µg/dL |  |  | | <55 |  | | ≥55-79.9 | |  | | ≥80-117.9 |  | | | ≥118 | | |  | | |  | | | | | |  | |
|  | No. cases/No. controls |  |  | | 94/205 |  | | 105/224 | |  | | 124/211 |  | | | 99/221 | | |  | | |  | | | | | |  | |
|  | all cancers |  |  | | 1.0 (referent) |  | | 1.0 (0.7-1.5) | |  | | 1.3 (0.9-1.8) |  | | | 1.0 (0.7-1.5) | | |  | | | 0.60 | | | | | |  | |
|  | invasive |  |  | | 1.0 (referent) |  | | 1.1 (0.8-1.7) | |  | | 1.3 (0.9-1.9) |  | | | 1.0 (0.6-1.5) | | |  | | | 0.86 | | | | | |  | |
|  | ER+/PR+ |  |  | | 1.0 (referent) |  | | 1.4 (0.9-2.2) | |  | | 1.7 (1.0-2.7) |  | | | 1.2 (0.8-2.1) | | |  | | | 0.24 | | | | | |  | |
|  |  |  |  | |  |  | |  | |  | |  |  | | |  | | |  | | |  | | | | | |  | |
| **SHBG** | |  |  | |  |  | |  | |  | |  |  | | |  | | |  | | |  | | | | | |  | |
|  | Cutpoints, nmol/L |  |  | | <45 |  | | ≥45-64.9 | |  | | ≥65-94.9 |  | | | ≥95 | | |  | | |  | | | | | |  | |
|  | No. cases/No. controls |  |  | | 82/208 |  | | 99/220 | |  | | 119/224 |  | | | 121/207 | | |  | | |  | | | | | |  | |
|  | all cancers |  |  | | 1.0 (referent) |  | | 1.2 (0.8-1.7) | |  | | 1.2 (0.9-1.8) |  | | | 1.4 (1.0-2.1) | | |  | | | 0.05 | | | | | |  | |
|  | invasive |  |  | | 1.0 (referent) |  | | 1.3 (0.8-1.9) | |  | | 1.4 (0.9-2.0) |  | | | 1.6 (1.1-2.4) | | |  | | | 0.03 | | | | | |  | |
|  | ER+/PR+ |  |  | | 1.0 (referent) |  | | 1.5 (0.9-2.4) | |  | | 1.6 (1.0-2.6) |  | | | 1.7 (1.1-2.8) | | |  | | | 0.03 | | | | | |  | |
|  |  |  |  | |  |  | |  | |  | |  |  | | |  | | |  | | |  | | | | | |  | |
| **POSTMENOPAUSAL AT DIAGNOSIS**** | | | | | | | | | |  |  | | |  | | |  | | | |  | | |  | | | | |  |
| **FOLLICULAR** | |  |  | |  |  | |  | |  | |  |  | | |  | | |  | | |  | | | | | |  | |
| **Estradiol** | |  |  | |  |  | |  | |  | |  |  | | |  | | |  | | |  | | | | | |  | |
|  | No. cases/No. controls |  |  | | 35/48 |  | | 19/38 | |  | | 20/44 |  | | | 18/46 | | |  | | |  | | | | | |  | |
|  | all cancers |  |  | | 1.0 (referent) |  | | 0.7 (0.3-1.6) | |  | | 0.9 (0.4-2.2) |  | | | 0.4 (0.2-1.2) | | |  | | | 0.14 | | | | | |  | |
|  |  |  |  | |  |  | |  | |  | |  |  | | |  | | |  | | |  | | | | | |  | |
| **Free Estradiol** | |  |  | |  |  | |  | |  | |  |  | | |  | | |  | | |  | | | | | |  | |
|  | No. cases/No. controls |  |  | | 36/44 |  | | 17/38 | |  | | 17/28 |  | | | 16/52 | | |  | | |  | | | | | |  | |
|  | all cancers |  |  | | 1.0 (referent) |  | | 0.6 (0.2-1.3) | |  | | 0.5 (0.2-1.5) |  | | | 0.2 (0.1-0.6) | | |  | | | <0.01 | | | | | |  | |
|  |  |  |  | |  |  | |  | |  | |  |  | | |  | | |  | | |  | | | | | |  | |
| **Estrone** | |  |  | |  |  | |  | |  | |  |  | | |  | | |  | | |  | | | | | |  | |
|  | No. cases/No. controls |  |  | | 32/59 |  | | 19/35 | |  | | 16/30 |  | | | 24/49 | | |  | | |  | | | | | |  | |
|  | all cancers |  |  | | 1.0 (referent) |  | | 1.2 (0.5-2.9) | |  | | 0.8 (0.3-2.3) |  | | | 1.0 (0.4-2.2) | | |  | | | 0.84 | | | | | |  | |
|  |  |  |  | |  |  | |  | |  | |  |  | | |  | | |  | | |  | | | | | |  | |
| **LUTEAL** | |  |  | |  |  | |  | |  | |  |  | | |  | | |  | | |  | | | | | |  | |
| **Estradiol** | |  |  | |  |  | |  | |  | |  |  | | |  | | |  | | |  | | | | | |  | |
|  | No. cases/No. controls |  |  | | 25/55 |  | | 22/45 | |  | | 26/48 |  | | | 25/42 | | |  | | |  | | | | | |  | |
|  | all cancers |  |  | | 1.0 (referent) |  | | 1.5 (0.6-4.2) | |  | | 1.3 (0.5-3.5) |  | | | 2.0 (0.8-5.1) | | |  | | | 0.20 | | | | | |  | |
|  |  |  |  | |  |  | |  | |  | |  |  | | |  | | |  | | |  | | | | | |  | |
| **Free Estradiol** | |  |  | |  |  | |  | |  | |  |  | | |  | | |  | | |  | | | | | |  | |
|  | No. cases/No. controls |  |  | | 23/53 |  | | 20/43 | |  | | 31/41 |  | | | 22/46 | | |  | | |  | | | | | |  | |
|  | all cancers |  |  | | 1.0 (referent) |  | | 1.4 (0.6-3.5) | |  | | 2.2 (1.0-5.1) |  | | | 1.1 (0.5-2.7) | | |  | | | 0.55 | | | | | |  | |
|  |  |  |  | |  |  | |  | |  | |  |  | | |  | | |  | | |  | | | | | |  | |
| **Estrone** | |  |  | |  |  | |  | |  | |  |  | | |  | | |  | | |  | | | | | |  | |
|  | No. cases/No. controls |  |  | | 29/59 |  | | 18/51 | |  | | 21/36 |  | | | 31/48 | | |  | | |  | | | | | |  | |
|  | all cancers |  |  | | 1.0 (referent) |  | | 0.7 (0.3-1.7) | |  | | 1.3 (0.6-3.1) |  | | | 1.5 (0.7-3.3) | | |  | | | 0.19 | | | | | |  | |
|  |  |  |  | |  |  | |  | |  | |  |  | | |  | | |  | | |  | | | | | |  | |
| **Progesterone** | |  |  | |  |  | |  | |  | |  |  | | |  | | |  | | |  | | | | | |  | |
|  | No. cases/No. controls |  |  | | 42/61 |  | | 16/37 | |  | | 17/37 |  | | | 21/58 | | |  | | |  | | | | | |  | |
|  | all cancers |  |  | | 1.0 (referent) |  | | 0.7 (0.3-1.8) | |  | | 0.6 (0.3-1.6) |  | | | 0.5 (0.2-1.3) | | |  | | | 0.16 | | | | | |  | |
|  |  |  |  | |  |  | |  | |  | |  |  | | |  | | |  | | |  | | | | | |  | |
| **LUTEAL AND UNTIMED** | |  |  | |  |  | |  | |  | |  |  | | |  | | |  | | |  | | | | | |  | |
| **Testosterone** | |  |  | |  |  | |  | |  | |  |  | | |  | | |  | | |  | | | | | |  | |
|  | No. cases/No. controls |  |  | | 42/73 |  | | 24/73 | |  | | 34/66 |  | | | 41/59 | | |  | | |  | | | | | |  | |
|  | all cancers |  |  | | 1.0 (referent) |  | | 0.5 (0.2-1.1) | |  | | 0.8 (0.4-1.6) |  | | | 1.2 (0.6-2.5) | | |  | | | 0.58 | | | | | |  | |
|  | invasive |  |  | | 1.0 (referent) |  | | 0.7 (0.3-1.6) | |  | | 1.4 (0.7-2.9) |  | | | 1.7 (0.8-3.6) | | |  | | | 0.07 | | | | | |  | |
|  | ER+/PR+ |  |  | | 1.0 (referent) |  | | 0.9 (0.4-2.1) | |  | | 1.5 (0.7-3.5) |  | | | 2.1 (0.9-5.0) | | |  | | | 0.05 | | | | | |  | |
|  |  |  |  | |  |  | |  | |  | |  |  | | |  | | |  | | |  | | | | | |  | |
| **Free Testosterone** | |  |  | |  |  | |  | |  | |  |  | | |  | | |  | | |  | | | | | |  | |
|  | No. cases/No. controls |  |  | | 33/68 |  | | 26/61 | |  | | 38/74 |  | | | 41/64 | | |  | | |  | | | | | |  | |
|  | all cancers |  |  | | 1.0 (referent) |  | | 0.9 (0.4-2.0) | |  | | 1.0 (0.5-2.1) |  | | | 1.5 (0.7-3.0) | | |  | | | 0.28 | | | | | |  | |
|  | invasive |  |  | | 1.0 (referent) |  | | 0.8 (0.4-1.7) | |  | | 0.9 (0.4-1.8) |  | | | 1.7 (0.8-3.4) | | |  | | | 0.15 | | | | | |  | |
|  | ER+/PR+ |  |  | | 1.0 (referent) |  | | 0.9 (0.4-2.1) | |  | | 0.9 (0.4-2.2) |  | | | 2.2 (1.0-5.0) | | |  | | | 0.06 | | | | | |  | |
|  |  |  |  | |  |  | |  | |  | |  |  | | |  | | |  | | |  | | | | | |  | |
| **DHEAS** | |  |  | |  |  | |  | |  | |  |  | | |  | | |  | | |  | | | | | |  | |
|  | No. cases/No. controls |  |  | | 35/77 |  | | 25/70 | |  | | 34/66 |  | | | 47/63 | | |  | | |  | | | | | |  | |
|  | all cancers |  |  | | 1.0 (referent) |  | | 0.7 (0.3-1.4) | |  | | 1.1 (0.5-2.3) |  | | | 1.6 (0.8-3.1) | | |  | | | 0.10 | | | | | |  | |
|  | invasive |  |  | | 1.0 (referent) |  | | 0.8 (0.4-1.6) | |  | | 1.1 (0.6-2.2) |  | | | 1.7 (0.9-3.4) | | |  | | | 0.10 | | | | | |  | |
|  | ER+/PR+ |  |  | | 1.0 (referent) |  | | 0.9 (0.4-2.2) | |  | | 1.6 (0.7-3.6) |  | | | 1.7 (0.7-3.8) | | |  | | | 0.15 | | | | | |  | |
|  |  |  |  | |  |  | |  | |  | |  |  | | |  | | |  | | |  | | | | | |  | |
| **SHBG** | |  |  | |  |  | |  | |  | |  |  | | |  | | |  | | |  | | | | | |  | |
|  | No. cases/No. controls |  |  | | 44/71 |  | | 37/70 | |  | | 33/65 |  | | | 24/64 | | |  | | |  | | | | | |  | |
|  | all cancers |  |  | | 1.0 (referent) |  | | 0.6 (0.3-1.2) | |  | | 0.8 (0.4-1.5) |  | | | 0.6 (0.3-1.2) | | |  | | | 0.18 | | | | | |  | |
|  | invasive |  |  | | 1.0 (referent) |  | | 0.9 (0.5-1.8) | |  | | 1.0 (0.5-2.0) |  | | | 0.8 (0.4-1.6) | | |  | | | 0.58 | | | | | |  | |
|  | ER+/PR+ |  |  | | 1.0 (referent) |  | | 0.7 (0.3-1.4) | |  | | 0.7 (0.3-1.6) |  | | | 0.6 (0.2-1.3) | | |  | | | 0.18 | | | | | |  | |
| All cancers: Conditional logistic regression for all cancers controlling for age at menarche, parity/age at first birth, BMI at age 18, family history of breast cancer, history of benign breast disease | | | | | | | | | | | | | | | | | | | | | | | | | |  | | | |
| Invasive and ER+/PR+ tumors: Unconditional logistic regression for invasive and ER+/PR+ disease controlling for factors listed above and matching factors. | | | | | | | | | | | | | | | | | | |  |  | | | | | | |  | | |
| *Quartiles cutpoints based on distributions of analyte in controls in overall population | | | | | | | | | | | | | | | | | | | | | | | | | |  | | | |
| **Data for invasive and ER+/PR+ disease not shown for estrogens and progesterone due to small case numbers in these subgroup for postmenopausal disease | | | | | | | | | | | | | | | | | | | | | | | | | |  | | | |
